# Cortical activation changes in supratentorial stroke patients during posture-cognition dual task

**DOI:** 10.3389/fneur.2025.1521687

**Published:** 2025-06-16

**Authors:** Jun He, Chen Gong, Xiang Zhu, Shizhe Zhu, Ayan Geng, Chaojie Kan, Sheng Xu, Tong Wang, Chuan Guo, Lan Zhu, Qinglei Wang

**Affiliations:** ^1^Department of Rehabilitation Medicine, Changzhou Dean Hospital, Changzhou, China; ^2^Department of Rehabilitation Medicine, The First Affiliated Hospital with Nanjing Medical University, Nanjing, China; ^3^School of Rehabilitation Medicine, Nanjing Medical University, Nanjing, China

**Keywords:** stroke, balance, dual task, cortical activation, fNIRS

## Abstract

**Objective:**

To explore the effects of postural control and cognition interference on cortical activation during balance tasks in stroke patients.

**Methods:**

fNIRS was used to measure cortical activation in the SMC, PMC, and PFC in 30 subjects with supratentorial stroke while performing a postural single task (PST), cognitive single task (CST), and postural-cognitive dual task (DT). Differences in activation and correlations with patient balance or cognitive performance were analyzed.

**Results:**

CST induced a higher level of activation in the unaffected SMC and bilateral PMC compared to PST. While DT resulted in more activation of the bilateral SMC and bilateral PMC compared to PST. No difference was found between DT and CST. Correlation analysis showed that activation of ROIs during balance tasks showed a positive correlation with the balance ability and cognitive performance of subjects.

**Conclusion:**

Both postural control and cognitive interference led to cortical activation changes during the tasks. Cognitive load was more likely to elicit greater cortical activation and approach the activation ceiling. These activations were intimately related to the patient’s ability to balance and cognitive performance. Subjects with better balance have a greater reserve of resources to allocate, enabling them to cope with tasks and improve task performance.

**Clinical trial registration:**

ClinicalTrials.gov. Identifier ChiCTR2300077916.

## 1 Introduction

Balance disorders are functional impairments that are prevalent following a stroke, with the highest prevalence rate reaching approximately 87.5% (1, 2). Even stroke patients with good functional recovery of the lower extremities also exhibit persistent impaired body sway control and postural control asymmetries (3). Patients with balance disorders commonly exhibit reduced balance self-efficacy and an increased fear of falling (4, 5). The challenges they meet not only impede their task performance but also significantly impact their quality of life, making it more difficult for them to return to normal community life (6, 7). One of the main reasons for this is that they face the dual difficulties of postural control and cognitive interference in their daily lives. Stroke patients are limited by impaired cortical resources, and the brain may exceed its central processing capacity when dealing with two tasks at the same time (8). When strokes patients are given a cognitive load, their motor performance tends to deteriorate and can even be accompanied by a decline in cognitive performance (9). Therefore, many studies use dual tasks (DTs) to explore the neural mechanisms related to balance and gait in stroke survivors. Such tasks can identify patients with balance disorders and at risk of falls and reveal ways to promote their recovery (10, 11).

Most previous studies using DTs have chosen walking as the designated motor task (12). When stroke patients perform dual-task walking, they usually exhibit increased gait variability and a decreased step speed (13, 14). This deterioration in dual-task ability tends to be more severe in stroke patients than in healthy subjects (15). However, dual-task postural control also needs to be assessed. The maintenance of a stable stance is a basic postural control requirement and is the foundation of the ability to walk. Many studies on postural control in stroke patients have focused on their level of control in response to postural perturbation tasks rather than cognitive interference (16, 17). Patients who have had a stroke experience many situations in their daily lives in which they must complete secondary tasks while maintaining their standing balance. Even for simple standing tasks, stroke patients present increased sway (18) and bilateral weight-bearing asymmetry (19), and much of their attention is focused on postural control (20) due to unilateral brain injury (21). This makes cognitive-motor interference a factor in postural control tasks. Bensoussan et al. (22) combined a simple standing task with a computational task and discovered that stroke patients had higher attentional demands for static postural control tasks than age-matched healthy subjects. Mehdizadeh et al. (23) measured the postural effects of a short-term memory task on different standing planes in stroke patients and found that increasing the task difficulty led to a decrease in cognitive and balance performance. Bhatt et al. (24) examined the degree to which semantic and working memory tasks interfered with balance control and observed that the stroke-related cognitive deficits may further significantly decrease the performance of dual task. Both authors quantified the degree of postural instability, but no studies have explored the associated central responses further. It remains unclear what changes occur in the cortex when cognitive interference is applied. In addition, several studies have applied dual-task training to improve postural stability in stroke patients, but the results achieved were inconsistent (25). Hence, measuring cortical activation during a standing balance task in the presence of cognitive interference may provide important insights into effective interventions (26, 27).

Functional near-infrared spectroscopy (fNIRS) is an advanced measurement tool that can help us to observe and measure cortical activation during balancing tasks (28). Using portable equipment, fNIRS monitors changes in cerebral hemodynamics during dynamic tasks in real time, and motion artifacts are easily accounted for Pinti et al. (29). Previous reports have used fNIRS to investigate stroke patients’ walking and balance control (30). Many brain regions have shown different activation levels and thus are likely to have important roles in postural control (17, 31). In studies using fNIRS monitoring of dual tasks, PFC was the most common region of focus, and this area has been strongly associated with cognitive and executive control (32). Studies have mainly found that it is activated in the presence of external perturbations in balance as well as during dual-task walking (33), and the activation of PFC correlates well with existing functional states (34). Following frontal lobe damage, patients have difficulty maintaining their postural balance during complex tasks (35). However, the changes related to postural control that occur under dual-task conditions in stroke survivors are not clear. The premotor cortex (PMC), anatomically localized between the anterior and posterior dorsolateral prefrontal cortex and primary motor cortex (M1), is another major cortical area involved in balance and movement (36). The PMC has been suggested to be involved in the temporal control and planning of locomotion and to play an important role in locomotor recovery after stroke (37). Liu et al. (38) drew a similar conclusion during a dual-task experiment, in which they observed a correlation between PMC activation and motor control performance. This is often observed in balance-related experiments but is less commonly addressed in dual-task studies. In addition, M1 and half of the primary sensory cortex are always in communication with the sensorimotor cortex (SMC), forming a major part of the motor cortex (39) that contributes to the higher-order control of motor behavior. Overall, the areas of brain that are active in stroke patients performing additional postural control or cognitive tasks have not been extensively explored. In addition, patients’ balance and cognition are closely related to the activation of brain regions (13, 40). Uncovering this partial association plays an important role in deepening our understanding of dual-task balance.

To address these issues, we added postural control requirements along with cognitive interference to the standing balance task, and fNIRS was used to observe the activation of brain regions related to balance and cognition during these processes. The activation patterns observed in stroke patients during balancing tasks will provide a reference for future assessments of balancing ability and the development of balance training programs.

## 2 Methods

### 2.1 Subjects

Thirty stroke subjects were recruited through publicity or medical record screening at the Rehabilitation Medicine Center of Changzhou Dean Hospital from August 2023 to December 2023. The inclusion criteria were as follows: (1) diagnosis of supratentorial stroke using computed tomography or magnetic resonance imaging; (2) first stroke located in a unilateral region; (3) unilateral limb paralysis; (4) 30–80 years of age; (5) disease duration between 1 month and 1 year; (6) mentally stable and cooperative, without evident cognitive impairments, and normal verbal functioning; (7) hemiplegic side of the lower limb meeting Brunnstrom III–V criteria; and (8) stance balance ≥Grade II (be capable of independently changing postures when standing and even resisting external disturbances) (41). The exclusion criteria were (1) cortical stroke; (2) suffering from progressive stroke or malignant progressive hypertension, severe visceral system diseases, malignant tumors, etc.; (3) previous history of organic brain diseases, mental disorders, or epilepsy; (4) presence of intracranial metal implantation or cranial defects; and (5) modified Ashworth score of the lower limb on the affected side ≥Grade III.

The experimental procedures were approved by the Human Ethics Committee of Dean Hospital of Changzhou (CZDALL-2023-013) and were performed in accordance with the ethical standards laid down in the 1975 Declaration of Helsinki (revised in 2008). The trial was registered at ClinicalTrials.gov (ChiCTR2300077916) on November 23, 2023.

### 2.2 Study design and experimental tasks

**Figure 1:**
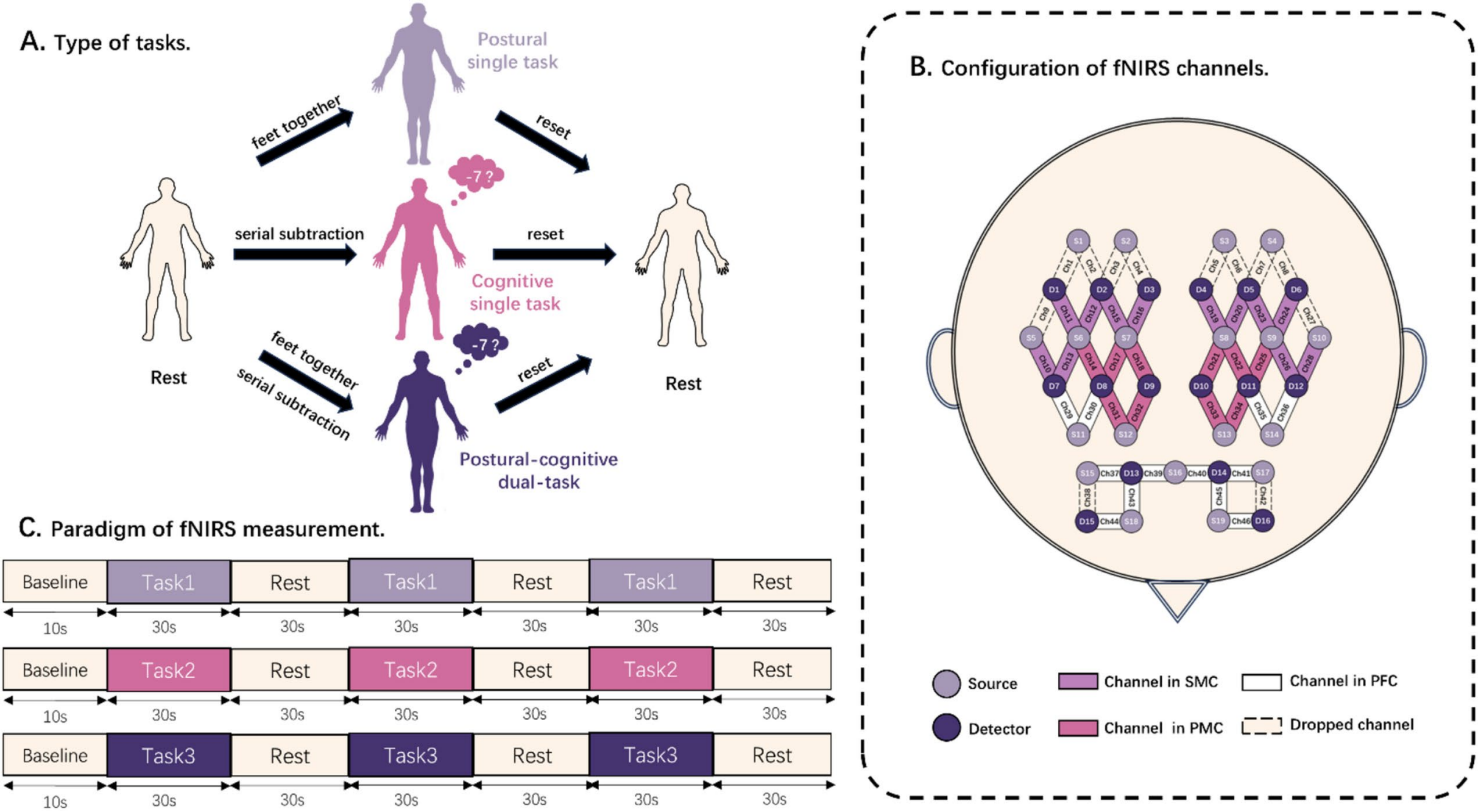
Diagram of balance task test. **(A)** Schematic representation of the components of the rest task and three balance tasks. **(B)** Distribution locations of all light sources and detectors. The channels are also divided into sensorimotor cortex (SMC), premotor cortex (PMC), and prefrontal cortex (PFC). **(C)** Paradigm of fNIRS acquisition. Task 1, Task 2, and Task 3 were randomized to postural single task, cognitive single task, and postural-cognitive dual task.

The subjects were required to adjust their posture, starting from the initial stance, to complete the following three balance tasks, (1) postural single task (PST), (2) cognitive single task (CST), and (3) postural-cognitive dual task (DT) (Figure 1A). For the initial stance, they were asked to stand quietly in an upright comfortable position while looking straight ahead. In the PST, subjects were instructed to comply with the therapist’s commands, moving the lower limbs from feet apart to feet together. In this posture, they should keep their heels close to the other heels and their toes close to the other toes until the end of the trial. It is worth noting that subjects needed to move the unaffected side to improve the efficiency and safety of the postural transitions. The CST consisted of serially subtracting 7 from a randomly chosen number between 200 and 300 with feet part in a regular standing posture. Subjects were asked to perform as many correct subtractions as possible while prioritizing correctness over speed, and the examiner counted the number of calculated answers and correct answers in each trial. The DT required the patient to perform a sequential calculation task with their feet together while the therapist recorded the number of calculated answers and correct answers. The accuracy and dual task cost is calculated by the following formula:


$$\text{Accuracy}=\frac{\text{The number of correct answers}}{\text{The number of calculated answers}}$$

$$\text{Dual task cost}=\frac{\text{Accuracy}\;(\mathrm{CST})-\text{Accuracy}\;(\mathrm{DT})}{\text{Accuracy}\;(\mathrm{CST})}$$

### 2.3 Balance ability assessment

The Berg Balance Scale (BBS), which consisted of 14 items, was used to assess the balance ability of the subjects. BBS is the most widely used scale in clinical practice for balance assessment (16). BBS can evaluate the ability of subjects to actively shift their center of gravity by observing a variety of functional activities, and permits a comprehensive examination of the dynamic and static balance of subjects in the sitting and standing positions. It was assessed by a trained therapist who was not aware of this study.

In addition, we used the Balance SD Dynamic and static balance test training system (Biodex, United States) to assess the ability of subjects to maintain stability. This system can objectively measure the balance performance of the subjects and make up for the deficiency that BBS is affected by subjectivity. Subjects were required to keep their center of gravity in a specified range as directed by the therapist. The evaluation index is the overall stability index (OSI). The OSI represents the displacement of the subject’s center of balance (COB) relative to the horizontal plane, recorded in degrees. The center of balance serves as the starting point for achieving an ideal state of balance. It can reflect the balance ability of the subject during the balance task objectively. Larger values indicate that the patient has a greater displacement of COB relative to the horizontal plane. And it reflects the poorer the balance ability.

### 2.4 fNIRS measurement

A continuous-wave fNIRS system (Nirsmart II, Danyang Huichuang Medical Equipment Co., Ltd., Zhenjiang, China), which is a 46-channel near-infrared spectroscopy detection system consisting of 19 light sources and 16 detector optodes, was used to measure changes in the concentration of oxygenated hemoglobin (HbO) and deoxygenated hemoglobin (HbR) in cerebral cortex. The distance between the sources and the detector optodes is 3 cm. The wavelengths of the NIR light sources were 730 and 850 nm, and a sampling rate of 11 Hz was used. An fNIRS test fiber optic cap was worn, and the CZ point of the 10/20 system of the internationally accepted EEG tracing method was used as the positioning point of the seventh source optode in the third row of the test optic caps. The light sources and the detector optodes were sequentially positioned to cover the bilateral PMC, SMC, and PFC, which were used as regions of interest (ROI). Channels that are not within the ROI will be dropped. All channels used for testing were within each ROI (Figure 1B).

The patient was positioned in a dimly lit, quiet room with the fNIRS cap on. The therapists introduced the three balancing tasks to fully familiarize the patient with them. Subjects performed 3 randomly ordered tasks successively: PST, CST, and DT. Prior to each measurement, the subjects were required to remain an initial stance as a rest for baseline measurements. Baseline data were collected for 10 s before each task. Afterward, a beep sounded, and a computerized voice told the patient to “start the task.” The patient then completed the corresponding balance task for 30 s. The computer sounded a beep and instructed the patient to “end the task.” The patient returned to the pre-task state, and they stayed standing and relaxed for 30 s. Then they waited for the next beep to begin another task. The whole process was repeated three times (Figure 1C). When measurements were completed for each task, the next task was assigned according to a previously randomized order. The rest interval between tasks was 5 min, during which time the patient sat down and rested to eliminate the effects of the previous task (15).

### 2.5 fNIRS data analysis

We used HbO as an indicator of the hemodynamic response, as it is more sensitive to changes in cortical activity than HbR (42). Data preprocessing was performed using the Homer2 toolbox of Matlab 2013b software. The preprocessing steps were as follows Brigadoi et al. (43). First, raw NIRS light intensity was converted to an optical density signal. Then we detected and corrected any motion artifacts caused by head motion during data acquisition using a motion artifact reduction algorithm, while the spline interpolation algorithm was used to correct for further motion artifacts. To remove the effects of physiological noise and drift, a low-pass bandpass filter with a frequency of 0.01–0.1 Hz was applied. Finally, the filtered optical density data were converted to HbO concentrations using the modified Beer–Lambert law. The hmrBlockAvg function was used to perform baseline corrections and to calculate the block averages for HbO changes over all trials and for each measurement channel. We chose 5 s prior to the trial onset as the baseline and 40 s after the trial onset to calculate the baseline-corrected block average. Following preprocessing, we used the baseline-corrected block averages to calculate the average values across a period of 5–35 s after trial onset due to the response delay of 3 to 4 s in the hemodynamic reaction (44). And they were defined as the ΔHbO of each trial. To distinguish the effects of the affected hemisphere from the unaffected hemisphere, we uniformly considered the right hemisphere as the affected hemisphere. For subjects with left hemispheric impact, the ΔHbO of the channel was flipped. Finally, the ΔHbO for all trials and all channels within the same ROI were averaged and used in the following statistical analysis.

### 2.6 Statistical analysis

The Shapiro–Wilk test was used to confirm the non-normal distribution of the basic characteristics and ΔHbO data. Paired *t*-tests were used to compare parameters consistent with normality. And the paired Wilcoxon test was used to compare parameters that did not conform to normality. Cortical activation data from the same ROI in three balance tasks were compared by the Friedman test. *Post-hoc* testing of pair-wise comparisons of cortical activation (i.e., PST vs. CST, PST vs. DT, and CST vs. DT) was then performed using the paired Wilcoxon test. The Spearman correlation test was then conducted to establish the potential correlations. For the above test, we also used false discovery rate correction for multiple comparisons across multiple brain regions and the three balance task conditions. The level of statistical significance for all analyses was set at *α* < 0.05.

## 3 Results

The characteristics of the participants are shown in Table 1. All subjects were able to perform all balancing tasks in the study successfully, and there were no adverse events such as falls during this study.

**Table 1:** Basic characteristics of the participants.

Age (years)	Gender (male/female)	Stroke type (I/H)	Post-stroke period (months)	Hemiparetic side (left/right)
56.500 ± 11.094	26/4	18/12	3.000 (1.750, 6.500)	15/15

Values are mean ± standard deviation, median (interquartile range) or ratio xx/xx. I, ischemic; H, hemorrhagic.

### 3.1 Balance and cognitive performance

The balance ability was reflected by the BBS and OSI. All included subjects had a BBS score of 43.000 (38.000, 47.250). And the OSI they measured was 1.000 (0.575, 1.225).

The cognitive performance of subjects is shown in Table 2. The number of calculated numbers performed by the patient in the DT was less than in the CST (*p* < 0.05), reflecting a decrease in the speed at which subjects counted subtractions. There was no significant change in the accuracy of calculations (*p* > 0.05).

**Table 2:** Cognitive performance in CST and DT.

Balance Task	Calculated number	Correct number	Accuracy	Dual task cost
CST	13.600 ± 6.652	12.767 ± 6.730	1.000 (0.857, 1.000)	
DT	12.433 ± 6.246	11.333 ± 6.326	0.932 (0.750, 1.000)	0.000 (−0.008, 0.150)
*t*/*Z*	2.827	2.979	−1.549	
*p*	0.008	0.006	0.121	

Values are mean ± standard deviation, and median (interquartile range). CST, cognitive single task; DT, postural-cognitive dual task.

### 3.2 Cortical activation

**Figure 2:**
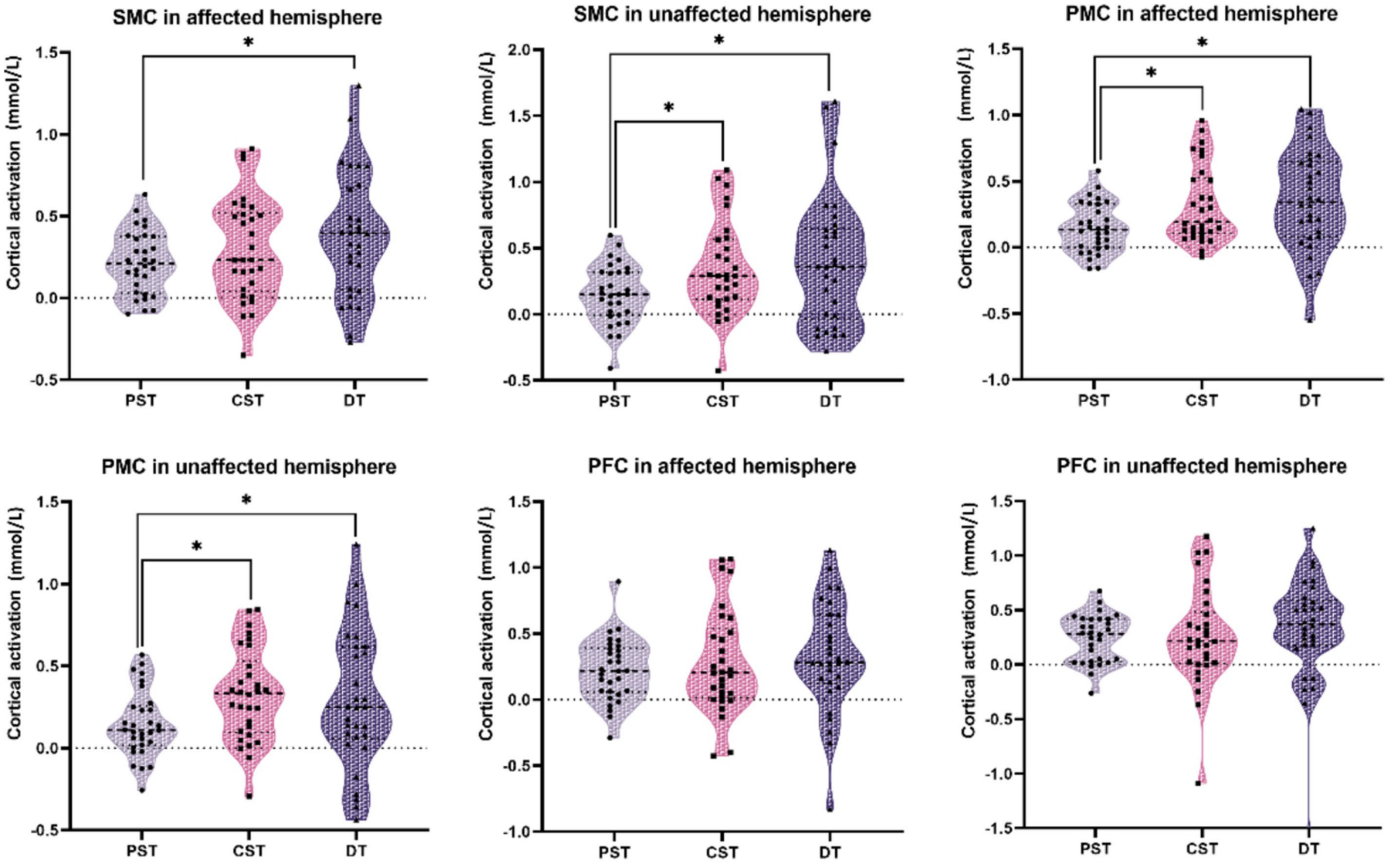
Comparison of cortical activation between two balance tasks. The black dots are the cortical activation values of each individual under this task and region of interest. Cortical activation was compared between the two groups (* indicates *p*_fdr_ < 0.05). PST, postural single task; CST, cognitive single task; DT, postural-cognitive dual-task; SMC, sensorimotor cortex; PMC, premotor cortex; PFC, prefrontal cortex.

The activation data for each ROI in the three tasks are shown in Table 3. We observed different activation patterns in the unaffected SMC, affected SMC, unaffected PMC, and affected PMC among the three tasks. No significant differences were found between the affected PFC and the unaffected PFC groups. The activation of unaffected SMC (*Z* = −2.437, *p*_fdr_ = 0.020), affected PMC (*Z* = −2.705, *p*_fdr_ = 0.014), and unaffected PMC (*Z* = −2.972, *p*_fdr_ = 0.012) during CST was higher than that during PST. The activation of affected SMC (*Z* = −2.314, *p*_fdr_ = 0.028), unaffected SMC (*Z* = −2.602, *p*_fdr_ = 0.019), affected PMC (*Z* = −2.808, *p*_fdr_ = 0.019), and unaffected PMC (*Z* = −1.985, *p*_fdr_ = 0.047) during DT was higher than that in PST. However, there was no significant difference in activation between CST and DT. The above results are shown in Figure 2.

**Table 3:** Cortical activation during three tasks.

ROI	PST (*N* = 30)	CST (*N* = 30)	DT (*N* = 30)	*χ* ^2^	*p*_fdr_
Affected SMC	0.210 (0.030, 0.376)	0.232 (0.042, 0.521)	0.395 (0.052, 0.673)	7.400	0.037
Unaffected SMC	0.015 (−0.007, 0.318)	0.289 (0.113, 0.566)	0.361 (−0.035, 0.647)	9.800	0.015
Affected PMC	0.133 (−0.001, 0.326)	0.192 (0.105, 0.524)	0.340 (0.080, 0.646)	13.400	0.007
Unaffected PMC	0.110 (0.016, 0.257)	0.332 (0.096, 0.531)	0.248 (0.056, 0.618)	10.067	0.015
Affected PFC	0.217 (0.057, 0.391)	0.205 (0.017, 0.537)	0.281 (0.125, 0.644)	1.800	0.407
Unaffected PFC	0.281 (0.025, 0.419)	0.217 (0.011, 0.483)	0.372 (0.176, 0.588)	6.067	0.058

ROI, region of interest; PST, postural single task; CST, cognitive single task; DT, postural-cognitive dual task; SMC, sensorimotor cortex; PMC, premotor cortex; PFC, prefrontal cortex.

### 3.3 Correlation analysis

In the three tasks, the balance ability of the subjects showed similar correlations with different ROIs (Table 4). During PST, the activations of unaffected SMC, affected SMC, unaffected PMC, and affected PMC were significantly and positively correlated with BBS but negatively correlated with OSI. During CST, the activations of unaffected PMC, affected PMC, unaffected PFC, and affected PFC were significantly and positively correlated with BBS but negatively correlated with OSI. Additionally, the activations of affected SMC and unaffected SMC were significantly and negatively correlated with OSI. During DT, the activations of unaffected SMC, affected SMC, unaffected PMC, affected PMC, unaffected PFC, and affected PFC were significantly and positively correlated with BBS but negatively correlated with OSI.

**Table 4:** Correlation between cortical activation and balance ability.

Balance task	ROI	Balance ability	Correlation coefficient	*p*	*p*_fdr_
PST	Affected SMC	BBS	0.454	0.012	0.023
		OSI	−0.404	0.027	0.044
	Unaffected SMC	BBS	0.459	0.011	0.023
		OSI	−0.404	0.027	0.044
	Affected PMC	BBS	0.492	0.006	0.023
		OSI	−0.170	0.368	0.039
	Unaffected PMC	BBS	0.382	0.037	0.048
		OSI	−0.219	0.246	0.277
	Affected PFC	BBS	0.247	0.188	0.199
		OSI	−0.044	0.819	0.819
	Unaffected PFC	BBS	0.268	0.152	0.171
		OSI	−0.236	0.210	0.252
CST	Affected SMC	BBS	0.337	0.069	0.083
		OSI	−0.534	0.002	0.036
	Unaffected SMC	BBS	0.240	0.201	0.201
		OSI	−0.380	0.038	0.049
	Affected PMC	BBS	0.446	0.013	0.023
		OSI	−0.439	0.015	0.044
	Unaffected PMC	BBS	0.554	0.001	0.018
		OSI	−0.423	0.020	0.044
	Affected PFC	BBS	0.534	0.002	0.018
		OSI	−0.438	0.015	0.044
	Unaffected PFC	BBS	0.469	0.009	0.023
		OSI	−0.411	0.024	0.044
DT	Affected SMC	BBS	0.411	0.024	0.036
		OSI	−0.471	0.009	0.044
	Unaffected SMC	BBS	0.382	0.037	0.048
		OSI	−0.393	0.032	0.048
	Affected PMC	BBS	0.448	0.013	0.023
		OSI	−0.385	0.036	0.049
	Unaffected PMC	BBS	0.463	0.010	0.023
		OSI	−0.411	0.024	0.044
	Affected PFC	BBS	0.484	0.007	0.023
		OSI	−0.475	0.008	0.044
	Unaffected PFC	BBS	0.431	0.017	0.028
		OSI	−0.452	0.012	0.044

ROI, region of interest; PST, postural single task; CST, cognitive single task; DT, postural-cognitive dual task; SMC, sensorimotor cortex; PMC, premotor cortex; PFC, prefrontal cortex; BBS, Berg Balance Scale; OSI, overall stability index.

For cognitive performance, the activations of unaffected SMC, affected PMC, un-affected PMC and affected PFC were significantly and positively correlated with the number of correct numbers during CST. The activations of all ROIs were significantly and positively correlated with the number of correct numbers during DT (Table 5).

**Table 5:** Correlation between cortical activation and cognitive performance.

Balance task	ROI	Cognitive performance	Correlation coefficient	*p*	*p*_fdr_
CST	Affected SMC	Correct number	0.339	0.067	0.080
		accuracy	−0.055	0.773	0.773
	Unaffected SMC	Correct number	0.496	0.005	0.015
		Accuracy	−0.106	0.577	0.692
	Affected PMC	Correct number	0.463	0.010	0.020
		Accuracy	−0.143	0.450	0.675
	Unaffected PMC	Correct number	0.539	0.002	0.012
		Accuracy	−0.215	0.254	0.675
	Affected PFC	Correct number	0.410	0.024	0.036
		accuracy	−0.176	0.352	0.675
	Unaffected PFC	Correct number	0.303	0.103	0.103
		Accuracy	−0.278	0.137	0.675
DT	Affected SMC	Correct number	0.471	0.009	0.014
		Accuracy	0.045	0.815	0.860
		Dual task cost	−0.097	0.609	0.695
	Unaffected SMC	Correct number	0.425	0.019	0.023
		Accuracy	0.034	0.860	0.860
		Dual task cost	−0.082	0.666	0.695
	Affected PMC	Correct number	0.542	0.002	0.012
		Accuracy	0.062	0.743	0.860
		Dual task cost	−0.075	0.695	0.695
	Unaffected PMC	Correct number	0.516	0.004	0.012
		Accuracy	0.270	0.149	0.860
		Dual task cost	−0.266	0.155	0.695
	Affected PFC	Correct number	0.468	0.009	0.014
		Accuracy	0.133	0.485	0.860
		Dual task cost	−0.183	0.333	0.695
	Unaffected PFC	Correct number	0.366	0.047	0.047
		Accuracy	0.063	0.740	0.860
		Dual task cost	−0.083	0.661	0.695

ROI, region of interest; CST, cognitive single task; DT, postural-cognitive dual task; SMC, sensorimotor cortex; PMC, premotor cortex; PFC, prefrontal cortex.

Additionally, correlation analyses of subjects’ balance ability with corresponding cognitive performance showed that OSI was negatively correlated with the number of correct answers during CST and DT (*p* < 0.05). No significant correlation was found be-tween cognitive performance and BBS (*p* > 0.05) (Table 6).

**Table 6:** Correlation between balance ability and cognitive performance.

Balance task	Balance ability	Cognitive performance	Correlation coefficient	*p*
CST	BBS	Correct number	0.236	0.210
		Accuracy	−0.154	0.416
	OSI	Correct number	−0.381	0.038
		Accuracy	−0.216	0.252
DT	BBS	Correct number	0.265	0.158
		Accuracy	0.113	0.550
		Dual task cost	−0.086	0.652
	OSI	Correct number	−0.387	0.035
		Accuracy	−0.288	0.123
		Dual task cost	0.082	0.667

CST, cognitive single task; DT, postural-cognitive; BBS, Berg Balance Scale; OSI, overall stability index.

## 4 Discussion

In contrast to previous studies of cortical activation in stroke patients during dual-task walking, we used a dual-tasking paradigm consisting of a cognitive task combined with a postural control task to explore the differences in cortical activation in patients given different balance tasks. We found that attempting posture control simultaneously with a single cognitive task resulted in higher cortical activation than a postural control only task in stroke subjects, and the pattern of activation more closely resembled that under a dual cognitive and postural load.

### 4.1 PST vs. CST

Generally, an increase in cognitive load or postural control requirements bring about an increase in the difficulty of the balancing task. First, standing with feet together can be a good postural control test, increasing the difficulty of static standing (45). As the support surface decreases, the patient’s standing instability increases. Hyndman et al. (46) indicated that, compared to the preferred posture, patients standing with their feet together had an increased sway index, which manifested in the medial lateral direction more than the anterior-posterior direction. Thus, patients must increase the degree of control over their bodies to avoid falling. When the difficulty of the task increases, brain regions tend to display increased activation that enhances their control of body posture (47). In our study, we observed the widespread increased activation of brain regions during the PST compared to the preferred stance, which is consistent with previous studies. Serial subtraction is a commonly used cognitive task paradigm in dual-task studies (48). Our previous study found that, among many types of dual-task paradigms, walking combined with serial subtraction more readily activates the PFC (49). Concurrently, in this study, we discovered more intense PFC activation in subjects performing the CST than the preferred stance, and the activation of other brain regions increased at the same time.

However, in the comparison with CST, we did not observe a significant difference in the PFC. This may have been related to the fact that the PFC is also intricately involved in the simple task of posture control, as evidence shows that PFC activation is required for upright posture in healthy humans (50). The increased activation of the PFC was also reported for a task that required more posture control but without cognitive tasks (51). The evidence suggested that PFC activation may be closely related to the relative increase in postural sway amplitude and frequency during unstable postures. The task of standing with feet together in our study similarly exacerbated the patients’ postural sway and resulted in the overactivation of the PFC. Another of our discoveries was the greater activation of motor-related cortical areas, such as the PMC and SMC, during CST compared to PST. A study that added either a motor task or a cognitive task to the original task, similar to the PST and CST in our study (38), also found higher PMC activation in the cognitive DT than the motor DT. Therefore, performing a second cognitive task while standing may require higher levels of attention and executive functioning than the PST and may interfere with postural control performance to a greater extent.

### 4.2 PST vs. DT

After adding further cognitive load to the PST, we found increased activation of the bilateral SMC and bilateral PMC, but no significant increase was found in the PFC. This differs from the results of previous dual-task walking studies, whose results typically show increased PFC activation when comparing DTs to single tasks. It is worth noting that most of these studies included chronic stroke patients with a disease duration usually greater than 6 months or even more than 1 year. A study exploring dual-task walking in subacute stroke patients found that PFC activity did not increase in dual tasking compared to single tasking, which is consistent with our results (15). Of the 30 subjects included in our study, only 7 had a disease duration of more than 6 months, while the others were in the subacute phase (<6 months) (52). This indicates that the brains of patients at this stage tend to follow a “postural prioritization” strategy. In the task prioritization strategy, a variety of factors influence the selection of tasks to be prioritized (53). Due to their poorer balancing ability, patients prioritize controlling their body to avoid the risk of falling when coping with difficult and complex tasks (54), and they allocate more resources to postural control. This explains why the activation of SMC and PMC was significantly higher in DT than PST in the present study. In addition, Liu et al. (38) observed changes in motor-related brain regions processing a single task versus a cognitive task and confirmed the activation of most of these brain regions. This suggests that a dose of cognitive load given to patients during training will help to improve the effectiveness of balance training. Moreover, a possible strategy to improve motor performance in stroke patients using motor or cognitive dual-task training may be to promote plasticity in the motor cortex.

### 4.3 CST vs. DT

When further postural control was required with the CST, there was no further activation of the brain regions. This phenomenon can be explained by the brain capacity model. Previous studies have pointed out that the capacity of the brain, which is analogous to the availability of resources, is limited (55). There may be a “ceiling” for the degree of cortical activation induced, which is mainly related to the resource competition and compensation mechanism (56). Stroke patients need to handle both cognitive and motor needs simultaneously in dual tasks, resulting in resource competition in brain. In addition, the cerebellum of the patients in our study was not affected. So, the cerebellum may also be involved and play an important compensatory role through the cerebellar-thalamus-brain circuit (57). They may compensate for the decline in function through excessive activation of bilateral brain regions, but this compensation fails at high task loads, manifested as the inability to further increase activation (58). In this case, the complex dual-task requirements cannot be dealt with. The interference effect will further increase and cause the deterioration of task performance (49). Brain hyperactivation in the poorly functioning stroke patients may have been triggered by the cognitive task of standing and could not be further enhanced by the additional demands of postural control. Alternatively, the postural control load may not have been sufficient to have a large impact on tasks involving an already existing cognitive load. In our study, it was also observed that subjects did not differ in the accuracy of answers when performing the DT compared to the CST. Although there was a difference in the number of answers, the DT was only about one less than the CST. It could be due to the delayed responses caused by the subjects’ simultaneous completion of the postural transitions as well as the calculations at the beginning. Cognitive performance may not be significantly affected during the actual subsequent sustained standing. Brown et al. (59) tested the behavioral performance of stroke survivors in a cognitive task versus a standing task. They found a significant difference in reaction time scores for combined cognitive tasks, but only between the sitting and side-by-side standing conditions, and no difference was found between standing and standing with feet together. This observation could be analogous to the comparison of PST and CST. The PST showed a low activation state compared to the CST.

### 4.4 Correlation with balance ability

We investigated the correlations between cortical activation in subjects and their balance abilities. In this study, stroke patients with better balance had a higher degree of SMC and PMC activation while performing PST, but there was no such correlation with PFC. It is understandable that SMC and PMC play important roles in purely postural balance standing tasks, as this has been shown in previous studies (12). The increased activation of the supplementary motor area (SMA) and SMC has also been observed during lower limb movement and gait recovery after stroke (60). Tests used to examine postural perturbations also revealed a significant positive correlation between SMA activation and BBS in stroke patients (40). These results further suggest that these regions are associated with postural balance recovery.

When a cognitive task component was present in the CST and DT, the activation of the PFC, besides the SMC and PMC, showed a significant correlation with the ability of balance. In addition, those subjects with better balance stability also had better cognitive performance. And the cognitive performance was also positively correlated with cortical activation in various brain regions. This could be explained by the fact that subjects with better balance may have more reserves of resources and allocate more re-sources to tasks to improve task performance. Subjects can flexibly adjust their priority strategies according to their own abilities. Therefore, this difference of activation may be a compensatory strategy for maintaining standing balance or an indicator of the upper limit of resources during recovery (40, 61). Helping patients to improve their task performance and promote their recovery through more active brain activity will be one of the strategies of rehabilitation in the future.

## 5 Limitations

Our study explored the feasibility of using fNIRS to measure cortical activation in stroke patients during a postural control task combined with a cognitive task in a dual-task paradigm. However, there were some limitations. First, a larger sample size could be included in the future to increase the reliability of the data. Second, there may be some correlation between brain area activation and current balance performance; however, studying this would require the patient to switch between two balance tasks during one test. This is not feasible with current balance-measuring instruments, as the patient’s center of gravity can change significantly with postural changes. Therefore, it is difficult to quantify balance performance under different balance tasks. In addition, patients in the chronic and subacute phases may exhibit different task strategies. This factor could not be differentiated in the present study. Furthermore, fNIRS can only measure the activation of the cortex. Due to the insufficient number of channels and the difficulty for near-infrared light to penetrate the thicker skull at this location. The parietal cortex and subcortical structures, especially the important structures involved in balance such as the cerebellum, have not been observed. It might ignore the crucial role of the cerebellum in tasks. Lastly, in future trials, more subjects in the chronic phase will be included, and subgroup analyses will be performed, to ascertain the differences brought about by their disease course. Meanwhile, we will control confounding factors such as drugs and underlying diseases that may affect the test results. Moreover, future investigations will employ functional magnetic resonance imaging to measure activation patterns in subcortical structures, particularly cerebellar and basal ganglia. This multimodal approach will allow comprehensive elucidation of the neurophysiological mechanisms underlying dual-task balance control, with specific emphasis on cortico-cerebellar interplay during postural challenge paradigms.

## 6 Conclusion

In this study, we observed cortical activation in supratentorial stroke patients under a situational balance task with postural control requirements, cognitive load, and both. The results suggested that the addition of cognitive interference while maintaining standing balance may require more brain area activation than the addition of postural control requirements. Moreover, PMC and SMC activity associated with movement were significantly involved in this process. However, the posture-cognition DT did not result in more prominent activation than the single cognitive load. This may reflect the existence of an activation ceiling that occurs in stroke patients when performing tasks. The extent of this activation may also be closely related to the balance ability and cognitive performance. This study has improved our understanding of the dual-tasking abilities of stroke patients. Further studies on stroke patient rehabilitation, including the optimum exercises to improve dual-tasking ability, and the effects of choosing more difficult postural balance tasks or different types of cognitive interference are warranted.

## Data Availability

The original contributions presented in the study are included in the article/Supplementary material, further inquiries can be directed to the corresponding authors.
