# Supplementary material for: Cortical activation changes in supratentorial stroke patients during posture-cognition dual task
Source: Front Neurol. 2025 Jun 16;16:1521687. doi: 10.3389/fneur.2025.1521687 (PMC12206779; doi:10.3389/fneur.2025.1521687)
Supplement: Supplementary file 1 [file Table_1.DOCX]

Supplementary Material

**Table1. Basic characteristics of the participants**

| **Sub** | **Age (years)** | **Sex** | **Stroke type** | **Post-stroke period (months)** | **Hemiparetic side** |
| --- | --- | --- | --- | --- | --- |
| 1 | 52 | M | I | 8 | L |
| 2 | 60 | M | H | 11 | R |
| 3 | 55 | F | H | 2 | L |
| 4 | 57 | M | H | 5 | L |
| 5 | 43 | M | I | 1 | L |
| 6 | 66 | M | I | 2 | R |
| 7 | 66 | M | I | 2 | R |
| 8 | 53 | M | H | 11 | R |
| 9 | 49 | M | H | 3 | R |
| 10 | 68 | M | I | 6 | L |
| 11 | 53 | M | H | 11 | R |
| 12 | 55 | F | I | 1 | L |
| 13 | 49 | M | H | 3 | R |
| 14 | 71 | M | H | 9 | R |
| 15 | 58 | F | H | 4 | R |
| 16 | 68 | M | I | 1 | L |
| 17 | 48 | M | I | 6 | L |
| 18 | 48 | M | I | 8 | R |
| 19 | 60 | M | I | 1 | L |
| 20 | 67 | M | I | 3 | R |
| 21 | 57 | M | I | 3 | L |
| 22 | 62 | M | H | 2 | R |
| 23 | 75 | M | I | 1 | L |
| 24 | 68 | M | I | 1 | L |
| 25 | 30 | M | I | 3 | L |
| 26 | 74 | M | I | 1 | R |
| 27 | 34 | M | H | 2 | L |
| 28 | 58 | F | I | 3 | L |
| 29 | 42 | M | I | 2 | R |
| 30 | 49 | M | H | 9 | R |

M, male; F, female; I, ischemic; H, hemorrhage; L, left; R, right.

**Table2. Comparison of brain activation between two balance tasks**

| ROI | affected SMC | | | unaffected SMC | | | affected PMC | | | unaffected PMC | | |
| --- | --- | --- | --- | --- | --- | --- | --- | --- | --- | --- | --- | --- |
|  | Z | P | P_fdr_ | Z | P | P_fdr_ | Z | P | P_fdr_ | Z | P | P_fdr_ |
| PST vs. CST | -1.574 | 0.116 | 0.116 | -2.437 | 0.015 | 0.020 | -2.705 | 0.007 | 0.014 | -2.972 | 0.003 | 0.012 |
| PST vs. DT | -2.314 | 0.020 | 0.028 | -2.602 | 0.009 | 0.019 | -2.808 | 0.005 | 0.019 | -1.985 | 0.047 | 0.047 |
| CST vs DT | -1.512 | 0.131 | 0.514 | -0.915 | 0.360 | 0.658 | -0.442 | 0.658 | 0.658 | 0.669 | 0.504 | 0.658 |

ROI, region of interest; PST, postural single-task; CST, cognitive single-task; DT, postural-cognitive dual-task; SMC, sensorimotor cortex; PMC, premotor cortex.

**Table3. The anatomical labels corresponding to the measurement channels.**

| Label CH | Brodmann Area (Chris rorden' MRIcro) | Percentage |
| --- | --- | --- |
| CH1 (S1-D1) | 7 - Somatosensory Association Cortex | 0.0281 |
|  | 39 - Angular gyrus_ part of Wernicke's area | 0.1486 |
|  | 40 - Supramarginal gyrus part of Wernicke's area | 0.8233 |
| CH2 (S1-D2) | 1 - Primary Somatosensory Cortex | 0.0041 |
|  | 2 - Primary Somatosensory Cortex | 0.0166 |
|  | 7 - Somatosensory Association Cortex | 0.6846 |
|  | 40 - Supramarginal gyrus part of Wernicke's area | 0.2946 |
| CH3 (S2-D2) | 1 - Primary Somatosensory Cortex | 0.0036 |
|  | 5 - Somatosensory Association Cortex | 0.0755 |
|  | 7 - Somatosensory Association Cortex | 0.9209 |
| CH4 (S2-D3) | 1 - Primary Somatosensory Cortex | 0.0853 |
|  | 5 - Somatosensory Association Cortex | 0.4505 |
|  | 7 - Somatosensory Association Cortex | 0.4642 |
| CH5 (S3-D4) | 1 - Primary Somatosensory Cortex | 0.0848 |
|  | 5 - Somatosensory Association Cortex | 0.3569 |
|  | 7 - Somatosensory Association Cortex | 0.5583 |
| CH6 (S3-D5) | 5 - Somatosensory Association Cortex | 0.0073 |
|  | 7 - Somatosensory Association Cortex | 0.9745 |
|  | 40 - Supramarginal gyrus part of Wernicke's area | 0.0182 |
| CH7 (S4-D5) | 7 - Somatosensory Association Cortex | 0.4873 |
|  | 40 - Supramarginal gyrus part of Wernicke's area | 0.5127 |
| CH8 (S4-D6) | 39 - Angular gyrus_ part of Wernicke's area | 0.2664 |
|  | 40 - Supramarginal gyrus part of Wernicke's area | 0.7336 |
| CH9 (S5-D1) | 1 - Primary Somatosensory Cortex | 0.2079 |
|  | 2 - Primary Somatosensory Cortex | 0.0323 |
|  | 40 - Supramarginal gyrus part of Wernicke's area | 0.7599 |
| CH10 (S5-D7) | 1 - Primary Somatosensory Cortex | 0.1832 |
|  | 3 - Primary Somatosensory Cortex | 0.2821 |
|  | 4 - Primary Motor Cortex | 0.3297 |
|  | 6 - Pre-Motor and Supplementary Motor Cortex | 0.1648 |
|  | 43 - Subcentral area | 0.0403 |
| CH11 (S6-D1) | 1 - Primary Somatosensory Cortex | 0.412 |
|  | 2 - Primary Somatosensory Cortex | 0.1536 |
|  | 3 - Primary Somatosensory Cortex | 0.2397 |
|  | 40 - Supramarginal gyrus part of Wernicke's area | 0.1948 |
| CH12 (S6-D2) | 1 - Primary Somatosensory Cortex | 0.1288 |
|  | 2 - Primary Somatosensory Cortex | 0.053 |
|  | 3 - Primary Somatosensory Cortex | 0.4432 |
|  | 4 - Primary Motor Cortex | 0.375 |
| CH13 (S6-D7) | 3 - Primary Somatosensory Cortex | 0.1976 |
|  | 4 - Primary Motor Cortex | 0.5403 |
|  | 6 - Pre-Motor and Supplementary Motor Cortex | 0.2621 |
| CH14 (S6-D8) | 3 - Primary Somatosensory Cortex | 0.0233 |
|  | 4 - Primary Motor Cortex | 0.5 |
|  | 6 - Pre-Motor and Supplementary Motor Cortex | 0.4767 |
| CH15 (S7-D2) | 1 - Primary Somatosensory Cortex | 0.0788 |
|  | 3 - Primary Somatosensory Cortex | 0.2534 |
|  | 4 - Primary Motor Cortex | 0.661 |
|  | 6 - Pre-Motor and Supplementary Motor Cortex | 0.0068 |
| CH16 (S7-D3) | 1 - Primary Somatosensory Cortex | 0.0423 |
|  | 3 - Primary Somatosensory Cortex | 0.2182 |
|  | 4 - Primary Motor Cortex | 0.7166 |
|  | 6 - Pre-Motor and Supplementary Motor Cortex | 0.0228 |
| CH17 (S7-D8) | 4 - Primary Motor Cortex | 0.0209 |
|  | 6 - Pre-Motor and Supplementary Motor Cortex | 0.9791 |
| CH18 (S7-D9) | 4 - Primary Motor Cortex | 0.0129 |
|  | 6 - Pre-Motor and Supplementary Motor Cortex | 0.9871 |
| CH19 (S8-D4) | 1 - Primary Somatosensory Cortex | 0.1367 |
|  | 3 - Primary Somatosensory Cortex | 0.2367 |
|  | 4 - Primary Motor Cortex | 0.6267 |
| CH20 (S8-D5) | 1 - Primary Somatosensory Cortex | 0.1375 |
|  | 2 - Primary Somatosensory Cortex | 0.0103 |
|  | 3 - Primary Somatosensory Cortex | 0.3024 |
|  | 4 - Primary Motor Cortex | 0.5498 |
| CH21 (S8-D10) | 4 - Primary Motor Cortex | 0.0099 |
|  | 6 - Pre-Motor and Supplementary Motor Cortex | 0.9901 |
| CH22 (S8-D11) | 4 - Primary Motor Cortex | 0.0696 |
|  | 6 - Pre-Motor and Supplementary Motor Cortex | 0.9304 |
| CH23 (S9-D5) | 1 - Primary Somatosensory Cortex | 0.2335 |
|  | 2 - Primary Somatosensory Cortex | 0.1673 |
|  | 3 - Primary Somatosensory Cortex | 0.393 |
|  | 4 - Primary Motor Cortex | 0.2062 |
| CH24 (S9-D6) | 1 - Primary Somatosensory Cortex | 0.4111 |
|  | 2 - Primary Somatosensory Cortex | 0.1186 |
|  | 3 - Primary Somatosensory Cortex | 0.1028 |
|  | 40 - Supramarginal gyrus part of Wernicke's area | 0.3676 |
| CH25 (S9-D11) | 3 - Primary Somatosensory Cortex | 0.021 |
|  | 4 - Primary Motor Cortex | 0.437 |
|  | 6 - Pre-Motor and Supplementary Motor Cortex | 0.542 |
| CH26 (S9-D12) | 3 - Primary Somatosensory Cortex | 0.1306 |
|  | 4 - Primary Motor Cortex | 0.4653 |
|  | 6 - Pre-Motor and Supplementary Motor Cortex | 0.4041 |
| CH27 (S10-D6) | 2 - Primary Somatosensory Cortex | 0.1728 |
|  | 40 - Supramarginal gyrus part of Wernicke's area | 0.8272 |
| CH28 (S10-D12) | 1 - Primary Somatosensory Cortex | 0.2647 |
|  | 3 - Primary Somatosensory Cortex | 0.261 |
|  | 4 - Primary Motor Cortex | 0.2794 |
|  | 6 - Pre-Motor and Supplementary Motor Cortex | 0.0662 |
|  | 43 - Subcentral area | 0.1287 |
| CH29 (S11-D7) | 6 - Pre-Motor and Supplementary Motor Cortex | 0.3171 |
|  | 9 - Dorsolateral prefrontal cortex | 0.6789 |
|  | 44 - pars opercularis_ part of Broca's area | 0.0041 |
| CH30 (S11-D8) | 6 - Pre-Motor and Supplementary Motor Cortex | 0.2055 |
|  | 8 - Includes Frontal eye fields | 0.2846 |
|  | 9 - Dorsolateral prefrontal cortex | 0.5099 |
| CH31 (S12-D8) | 6 - Pre-Motor and Supplementary Motor Cortex | 0.2721 |
|  | 8 - Includes Frontal eye fields | 0.7032 |
|  | 9 - Dorsolateral prefrontal cortex | 0.0247 |
| CH32 (S12-D9) | 6 - Pre-Motor and Supplementary Motor Cortex | 0.8157 |
|  | 8 - Includes Frontal eye fields | 0.1843 |
| CH33 (S13-D10) | 6 - Pre-Motor and Supplementary Motor Cortex | 0.7324 |
|  | 8 - Includes Frontal eye fields | 0.2676 |
| CH34 (S13-D11) | 6 - Pre-Motor and Supplementary Motor Cortex | 0.2372 |
|  | 8 - Includes Frontal eye fields | 0.6788 |
|  | 9 - Dorsolateral prefrontal cortex | 0.0839 |
| CH35 (S14-D11) | 6 - Pre-Motor and Supplementary Motor Cortex | 0.3074 |
|  | 8 - Includes Frontal eye fields | 0.1844 |
|  | 9 - Dorsolateral prefrontal cortex | 0.5082 |
| CH36 (S14-D12) | 6 - Pre-Motor and Supplementary Motor Cortex | 0.2407 |
|  | 9 - Dorsolateral prefrontal cortex | 0.6556 |
|  | 44 - pars opercularis_ part of Broca's area | 0.1037 |
| CH37 (S15-D13) | 9 - Dorsolateral prefrontal cortex | 0.0657 |
|  | 45 - pars triangularis Broca's area | 0.0751 |
|  | 46 - Dorsolateral prefrontal cortex | 0.8592 |
| CH38 (S15-D15) | 45 - pars triangularis Broca's area | 0.5376 |
|  | 46 - Dorsolateral prefrontal cortex | 0.4624 |
| CH39 (S16-D13) | 9 - Dorsolateral prefrontal cortex | 0.3918 |
|  | 10 - Frontopolar area | 0.6045 |
|  | 46 - Dorsolateral prefrontal cortex | 0.0037 |
| CH40 (S16-D14) | 9 - Dorsolateral prefrontal cortex | 0.3846 |
|  | 10 - Frontopolar area | 0.6154 |
| CH41 (S17-D14) | 45 - pars triangularis Broca's area | 0.0349 |
|  | 46 - Dorsolateral prefrontal cortex | 0.9651 |
| CH42 (S17-D16) | 45 - pars triangularis Broca's area | 0.5323 |
|  | 46 - Dorsolateral prefrontal cortex | 0.4677 |
| CH43 (S18-D13) | 10 - Frontopolar area | 0.8229 |
|  | 46 - Dorsolateral prefrontal cortex | 0.1771 |
| CH44 (S18-D15) | 10 - Frontopolar area | 0.6062 |
|  | 46 - Dorsolateral prefrontal cortex | 0.3938 |
| CH45 (S19-D14) | 10 - Frontopolar area | 0.993 |
|  | 46 - Dorsolateral prefrontal cortex | 0.007 |
| CH46 (S19-D16) | 10 - Frontopolar area | 0.7188 |
|  | 11 - Orbitofrontal area | 0.1523 |
|  | 46 - Dorsolateral prefrontal cortex | 0.125 |
|  | 47 - Inferior prefrontal gyrus | 0.0039 |
